# Mechanisms Underlying the Risk to Develop Drug Addiction, Insights From Studies in *Drosophila melanogaster*

**DOI:** 10.3389/fphys.2018.00327

**Published:** 2018-04-24

**Authors:** Julia Ryvkin, Assa Bentzur, Shir Zer-Krispil, Galit Shohat-Ophir

**Affiliations:** The Mina & Everard Goodman Faculty of Life Sciences and The Leslie and Susan Gonda Multidisciplinary Brain Research Center, Bar-Ilan University, Ramat Gan, Israel

**Keywords:** *Drosophila melanogaster*, reward, ethanol, addiction, learning and memory, natural reward, drug reward, animal models

## Abstract

The ability to adapt to environmental changes is an essential feature of biological systems, achieved in animals by a coordinated crosstalk between neuronal and hormonal programs that allow rapid and integrated organismal responses. Reward systems play a key role in mediating this adaptation by reinforcing behaviors that enhance immediate survival, such as eating or drinking, or those that ensure long-term survival, such as sexual behavior or caring for offspring. Drugs of abuse co-opt neuronal and molecular pathways that mediate natural rewards, which under certain circumstances can lead to addiction. Many factors can contribute to the transition from drug use to drug addiction, highlighting the need to discover mechanisms underlying the progression from initial drug use to drug addiction. Since similar responses to natural and drug rewards are present in very different animals, it is likely that the central systems that process reward stimuli originated early in evolution, and that common ancient biological principles and genes are involved in these processes. Thus, the neurobiology of natural and drug rewards can be studied using simpler model organisms that have their systems stripped of some of the immense complexity that exists in mammalian brains. In this paper we review studies in *Drosophila melanogaster* that model different aspects of natural and drug rewards, with an emphasis on how motivational states shape the value of the rewarding experience, as an entry point to understanding the mechanisms that contribute to the vulnerability of drug addiction.

## Introduction

From insects to humans, organisms living in complex environments need to respond quickly and appropriately to different stimuli by choosing one action over another to increase their chances of survival and reproduction. Reward systems play a key role in promoting this aim by motivating animals to repeat behaviors that increase their fitness, such as eating, drinking, sexual interaction, and parental behaviors. Drugs of abuse affect the same brain regions used for the processing of natural rewards, creating the pleasurable feeling indicative of a fitness benefit, and with repeated use can lead to compulsive drug abuse and addiction (Nesse and Berridge, 1997; Koob, 2009).

The American Psychiatric Association defines addiction as “maladaptive pattern of substance use manifested by recurrent and significant adverse consequences related to the repeated use of substances” (American Psychatric Association, 2013). This is characterized by a sequence of stages: (1) initial voluntary consumption of the drug, accompanied by an acute hedonic drug response, (2) repeated use, leading to compulsive and uncontrolled drug use, and finally, (3) physical and mental dependence (Koob and Bloom, 1988; Wolffgramm and Heyne, 1995; Koob, 1997, 2009; Nesse and Berridge, 1997).

Understanding the complex nature of human addiction is one of the greatest challenges in contemporary neuroscience, requiring parallel efforts of many scientific disciplines. One important approach is the use of animal systems to model certain features of the process, such as the reinforcing properties of drug rewards. Early studies by Karl von Frisch demonstrated the ability of sugar reward to reinforce preference for certain colors in honey bees (von Frisch, 1914). Subsequent studies by Olds and Milner demonstrated that rodents can learn to press a lever to receive intracranial self-stimulation (ICSS), facilitating the discovery of brain areas that encode reward (Olds and Milner, 1954). These seminal studies paved the path for the development of complex behavioral paradigms that measure the rewarding effects of drugs. Examples include self-administration paradigms, in which voluntary lever pressing results in delivery of a drug dose (Weeks, 1962; Thompson and Schuster, 1964), and conditioned place preference, where animals learn to associate a certain environment with receiving a drug, and the preference for this environment is tested afterwards in the absence of the drug (Rossi and Reid, 1976). Although the existing models do not entirely recapitulate the complexity of human addiction, they model important features of drug addiction (Koob, 2009; Lynch et al., 2010). For example, the positive reinforcing actions of binge intoxication is captured using self-administration paradigms in rodents and monkeys (Johanson and Balster, 1978; Collins et al., 1984), while the negative reinforcing properties of the withdrawal phase are measured by increased anxiety-like responses (Sanchis-Segura and Spanagel, 2006). The craving stage can be modeled by “cue-induced reinstatement,” in which the reinstatement of drug seeking is tested after the induction of drug cues following drug self-administration training (Sanchis-Segura and Spanagel, 2006; Liu et al., 2008; Mantsch et al., 2016).

**Figure 1:**
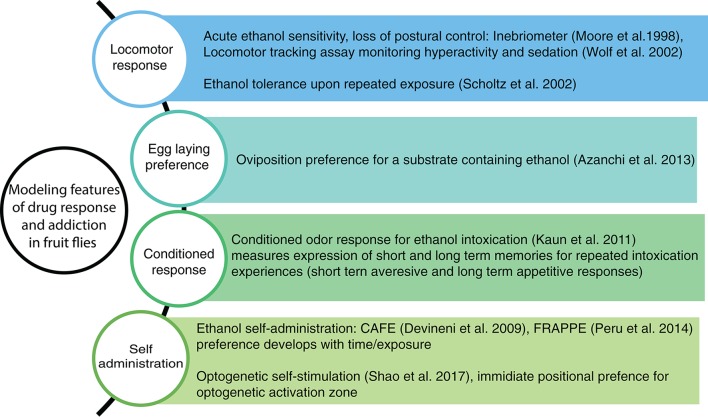
Schematic illustration of the behavioral paradigms that are used to model different features of drug addiction in *Drosophila*.

Although it is more common to use mammals to study addiction, insect behavior is no less organized and driven by reward. Many studies over the years have established the fruit fly *Drosophila melanogaster* as a non-conventional but very relevant model to explore molecular mechanisms underlying drug response. These have mostly focused on ethanol, modeling early stages of ethanol exposure, including its immediate locomotor effects (reviewed extensively in Rodan and Rothenfluh, 2010; Kaun et al., 2012; Devineni and Heberlein, 2013; Ghezzi et al., 2013a), its hedonic value, as reflected by voluntary consumption (Devineni and Heberlein, 2009), and the formation of long-lasting memories for the rewarding experience (Kaun et al., 2011; Figure 1). This review will present recent progress in which fruit flies were used to uncover genetic and environmental elements that influence the likelihood of progressing from initial exposure to repeated drug use. It will focus on drug-oriented studies and those that are not drug oriented but share mutual mechanisms and principles with addiction, such as learning and memory, and neuronal mechanisms that encode and process natural rewards. Together, the cellular pathways, neuronal circuits and newly discovered principles that govern reward processing can serve as a conceptual framework for understanding the mechanisms that underlie the risk to develop addiction.

## Methods of studying ethanol related behaviors in fruit flies

Flies encounter ethanol in their natural habitat, and as such, acquired many adaptations that enable them to survive and thrive in ethanol-rich environments (Gibson et al., 1981). Flies exhibit natural preference for ethanol: the smell of ethanol was shown to be an attractive cue using olfactory trap (Reed, 1938; Dudley, 2002; Devineni and Heberlein, 2009), and females show preference to lay eggs on ethanol containing substrates (Siegal and Hartl, 1999; Azanchi et al., 2013; Kacsoh et al., 2013). Flies develop preference to consume ethanol-containing food in a two-choice consumption paradigm. The kinetics of their preference and its extent depend on genetic background (Merçot et al., 1994; Devineni and Heberlein, 2009), prior exposure to ethanol (Peru y Colón de Portugal et al., 2014), sampling time (Devineni and Heberlein, 2009), and prior sexual experience (Shohat-Ophir et al., 2012). Importantly, flies display similar behavioral responses to acute exposure to ethanol as mammals: increased motor response when exposed to a low dose of ethanol, and sedation when reaching higher doses (Singh and Heberlein, 2000). Repeated exposure to ethanol results in functional tolerance and increases the time and dose needed to induce sedation. This reflects neuronal plasticity that corresponds to tolerance (Figure 1), but can also be caused by changes in ethanol metabolism (Scholz et al., 2000).

Over the years there have been several experimental systems to study the locomotor response to ethanol intoxication, the first of which was the inebriometer system (Cohan and Graf, 1985; Cohan and Hoffmann, 1986; Weber, 1988), in which flies lose their postural control when exposed to ethanol vapor. The system was later adapted for high throughput functional genetic screens by the Heberelin lab (Moore et al., 1998), and was subsequently replaced by video tracking systems that measure changes in fly velocity during acute intoxication, and assays that measure loss of righting response when reaching sedating levels (Wolf et al., 2002; Maples and Rothenfluh, 2011). Many genes and cellular pathways in neurons and glia cells have been shown to modulate the sensitivity of flies to both the positive and negative motor responses upon exposure to ethanol vapor, and the development of tolerance (Moore et al., 1998; Scholz et al., 2000, 2005; Berger et al., 2004; Ghezzi et al., 2004, 2013b; Corl et al., 2005; Cowmeadow et al., 2006; Kong et al., 2010; King et al., 2011, 2014; Kapfhamer et al., 2012; Krishnan et al., 2012, 2016; Devineni et al., 2013; Li et al., 2013; McClure and Heberlein, 2013; Pohl et al., 2013; Troutwine et al., 2016). Some of the identified fly genes, pathways and principles paved the way for parallel studies in mammals (Corl et al., 2009; Lasek et al., 2011a,b,c; Maiya et al., 2012, 2015; Kapfhamer et al., 2013).

A breakthrough in modeling aspects of drug reward in flies was the introduction of two paradigms: a conditioned response to ethanol vapor (Kaun et al., 2011), and a two-choice assay to measure voluntary ethanol consumption (Ja et al., 2007; Devineni and Heberlein, 2009). In the first paradigm, flies learn to associate cues with ethanol intoxication and develop long-lasting attraction for an ethanol-paired cue (Kaun et al., 2011). A demonstration for the relevance of this model as a system to study aspects of drug reward was the finding that flies are willing to tolerate electric shock in order to approach an odor cue predicting ethanol reward (Kaun et al., 2011). The two-choice ethanol consumption paradigm measures motivation to obtain drug rewards, where flies can choose to feed from ethanol or non-ethanol containing food in a capillary feeder system (CAFE) (Devineni and Heberlein, 2009; Pohl et al., 2012; Shohat-Ophir et al., 2012; Xu et al., 2012; Ojelade et al., 2015; Zer et al., 2016). Another two-choice ethanol consumption paradigm is the FRAPPE (Fluorometric Reading Assay of Preference Primed by Ethanol), a novel assay based on the CAFE system, which allows precise and high throughput measurement of consumption in individual flies (Peru y Colón de Portugal et al., 2014; Figure 1). Lastly, a recent study by Shao, et al. established a new reward self-administration paradigm that is based on optogenetic stimulation of neurons that encode positive valence (Shao et al., 2017). In this assay, flies harboring the red shifted channel rhodopsin CsChrimson (Inagaki et al., 2014a) in NPF neurons prefer to be in a zone that triggers optogenetic stimulation of their NPF expressing neurons (Shao et al., 2017; Figure 1). Although this assay does not measure drug related responses, it facilitates the identification of neurons that induce immediate pleasure, and conceptually resembles the rodent intracranial self-stimulation (ICSS) paradigm (Olds and Milner, 1954).

## Drug-unrelated studies and their contribution to understanding the molecular basis of addiction: the case of learning and memory

Addiction is frequently referred to as pathological usurpation of learning and memory mechanisms that are normally used to predict the occurrence of natural rewards (Nestler, 2002; Hyman, 2005; Hyman et al., 2006; Kalivas and O'Brien, 2008; Duan et al., 2016; Patrono et al., 2016). This part of the review will explore the contribution of the field of learning and memory in flies to understanding drug related behaviors and possibly addiction, by covering two major directions in the field: traditional forward genetic screens, and more recent circuitry-oriented studies.

### Genes and cellular pathways that constitute the basic machinery encoding learning and reward

Seymour Benzer and colleagues were the first to demonstrate that one can use a genetic scalpel to identify genes and pathways that are necessary for the formation of memory (Quinn et al., 1974). Learning and memory can be studied in flies using both reward or avoidance of punishment based assays, by pairing a neutral cue to the presence of sucrose (positive reinforcement) or electric shock (punishment) (Quinn et al., 1974; Tempel et al., 1983). The memory for the experience is measured by testing the avoidance or attraction of the flies to the odor that was previously paired (conditioned stimulus) with the experience (unconditioned stimulus). Many studies over the years identified mutants in different stages of the process, some of which showed virtually no learning during shock training, like the mutant *turnip* (Quinn et al., 1979), *dunce* (Dudai et al., 1976), and *rutabaga* (Aceves-Piña and Quinn, 1979), while others learned normally but forgot the task of shock and sucrose training faster than wild type flies, like *amnesiac* (Quinn et al., 1979; Tempel et al., 1983). The elucidation of the molecular functions of the affected genes shed light on the biochemical mechanisms mediating learning and memory, and indicated a pivotal function for the cAMP pathway; *rutabaga (rut)* encodes for the Ca^2+^/CaM-sensitive adenylyl cyclase (Livingstone et al., 1982), and *Dunce* has cAMP phosphodiesterase activity (Byers et al., 1981). In addition to the cAMP pathways, other studies identified additional players that regulate memory related plasticity events, such as Ca^2+^/CaM Kinase II (Joiner and Griffith, 1997) and Orb2, a CPEB protein that functions in synaptic plasticity-required protein synthesis (Keleman et al., 2007).

### Studying neuronal circuits that encode associative learning; the mushroom bodies as an association center

Recent technological advances in neurogenetics led to the emergence of powerful genetic tools such as optogenetics, *in vivo* Ca^2+^ imaging, and the ability to manipulate single neurons in behaving animals. This resulted in an explosion of studies on mechanisms that encode associative learning and the processing of natural rewards (reviewed by Owald et al., 2015). A central player in integrating the conditioned and unconditioned stimuli of a given experience into an associative memory is the Mushroom Body (MB), a brain region extensively studied with classical conditioning assays and genetic manipulations (Heisenberg et al., 1985; Connolly et al., 1996; Wolf et al., 1998; Waddell et al., 2000; McGuire et al., 2001; Liu et al., 2007; Thum et al., 2007; Aso et al., 2009) (reviewed by Kaun and Rothenfluh, 2017; Cognigni et al., 2018). Below we introduce some basic principles that govern the function of the MB, as an introduction to the neuronal machinery that processes positive reinforcement, which is required for reward learning. As such, this is not intended to be a comprehensive review of the MB [for detailed up to date reviews on the wiring and function of the MB see (Scaplen and Kaun, 2016; Felsenberg et al., 2017; Kaun and Rothenfluh, 2017; Cognigni et al., 2018)].

The MB is a brain area where visual (Vogt et al., 2014), gustatory (Kirkhart and Scott, 2015), thermal (Yagi et al., 2016), and olfactory (Stocker et al., 1990; Wong et al., 2002; Tanaka et al., 2004; Liu et al., 2008, 2012; Caron et al., 2013) information (conditioned stimuli) reaches a set of intrinsic neurons called Kenyon Cells (KC). KC axons run in parallel through MB lobes and synapse with different subsets of Mushroom Body Output Neurons (MBON) (Takemura et al., 2017), forming functionally segregated compartments. MBONs integrate sensory information with the valence of the experience (Hige et al., 2015), generating an association between the conditioned and unconditioned stimuli, and leading to associative memory formation. For this to happen, specific sub-populations of Dopaminergic Neurons (DAN) that innervate each compartment deliver information about the valence of the experience (unconditioned stimulus) (Thum et al., 2007; Aso et al., 2010, 2014a,b; Liu et al., 2012; Caron et al., 2013; Clowney et al., 2015) (reviewed by Das et al., 2016).

Activation of different populations of DANs is sufficient for aversive or appetitive memory formation when paired with a CS (reviewed by Waddell, 2013). Further functional dissections revealed that different subpopulations of DANs and MBONs encode information regarding the sweet vs. caloric value of the ingested food (Das et al., 2014), water reward (Shyu et al., 2017), aversive taste (Masek et al., 2015), electric shock memory (Unoki et al., 2005; Aso et al., 2010), and even specific short and long-term memory formation (Aso et al., 2014b). Memory formation, consolidation, retrieval, reconsolidation and/or extinction have been shown to occur via neuronal activities in specific parts of the MBONs and specific subsets of reinforcing DANs (Berry et al., 2012, 2015; Shuai et al., 2015; Aso and Rubin, 2016; Ichinose and Tanimoto, 2016) reviewed by Cognigni et al. (2018). Intriguingly, re-evaluation of previously learned appetitive memory was shown to be conveyed by the activity of a subset of MBONs that is anatomically connected to both aversive and appetitive DANs, and that extinction or re-consolidation of appetitive memories requires activity of both during re-evaluation (Felsenberg et al., 2017). Finally, a recent comprehensive connectome map of the entire MB alpha lobe that was generated by electron microscopy imaging, demonstrated that the interconnectivity between KCs, DANs and MBONs is even more intricate than previously thought, paving the path for further delineation of the underlying neurobiological principles of this brain region (Takemura et al., 2017).

### Shared molecular machinery of memory, reward, and drug-related behaviors in model organisms

Drug rewards converge on molecular and neural pathways that encode memory for natural rewards, and induce similar neuroplastic changes as natural rewards (reviewed by Hyman et al., 2006; Kauer and Malenka, 2007; Kalivas and O'Brien, 2008; Koob and Volkow, 2010). The cAMP, CREB dependent and ΔFosB pathways play a prominent role in mediating these long-term adaptive changes in neuronal function (Nestler, 2002; Mameli and Lüscher, 2011). An example of the crosstalk between natural reward, drug reward and neuroplasticity is demonstrated in studies where periods of abstinence from sexual experience increase the sensitization of rats to amphetamine reward (Bradley and Meisel, 2001; Pitchers et al., 2010). This sex experience-induced plasticity, which in turn causes enhanced drug reward, was shown to be mediated by dopamine 1 receptor (D1R)-dependent induction of ΔFosB in the nucleus accumbens (NAc) (Pitchers et al., 2013). A similar phenomenon was also documented in *Drosophila*, in which sexual deprivation increased the motivation to consume ethanol as a drug reward, by regulating the brain levels of neuropeptide F (NPF) (Shohat-Ophir et al., 2012).

As stated previously, the dopaminergic system plays a central role in processing natural rewards, and represents one way by which drugs of abuse induce changes in memory-related mechanisms (Di Chiara, 1999). In mammals, dopaminergic neurons show characteristic burst-firing activity during mating and food consumption (Dackis and O'Brien, 2001). Cocaine increases dopaminergic neurotransmission by blocking dopamine transport, preventing its removal from the synaptic cleft (Dackis and O'Brien, 2001). Reducing dopamine levels in fruit flies, using a competitive agonist to tyrosine hydroxylase (which converts tyrosine to L-Dopa), diminishes their sensitivity to cocaine and nicotine (Bainton et al., 2000). Dopamine release is also required for the expression of ethanol reward in fruit flies, as temporal block of neurotransmission in dopaminergic neurons prevented conditioned preference for ethanol-associated cues (Kaun et al., 2011). In addition, artificial activation of a certain dopamine neurons such as the protocerebral anterior medial (PAM neurons) is rewarding *per se*, as it induces robust appetitive odor memory in the absence of natural or drug reward (Liu et al., 2012).

### Shared mechanisms for ethanol-related behaviors and learning and memory in flies

Examining the connection between neuroplasticity and drug response, several studies tested whether established learning and memory mutants also depict aberrant behavioral phenotypes in acute ethanol response. The mutant *cheapdate*, which is an allele of the memory mutant *amnesiac*, caused increased sensitivity to the sedating effects of ethanol (Moore et al., 1998; Wolf and Heberlein, 2003). Another mutant, *rut*, exhibited increased ethanol hyperactivity and sensitivity (Wolf et al., 2002; Heberlein et al., 2004). In addition to acute responses to ethanol, learning and memory mutants revealed altered rapid and chronic tolerance responses to ethanol (for detailed list of genes see (Berger et al., 2008). For instance, the long-term memory mutant *john* displayed enhanced chronic tolerance in response to prolonged exposure (20–28 h) to low concentration of ethanol vapor (Berger et al., 2008).

*Krasavietz* (or *exba*), which encodes a translation initiation factor, is an example of a gene involved in learning and memory whose mutation affects both acute ethanol response and the motivation to consume ethanol. *Krasavietz* mutant flies exhibit decreased sensitivity to ethanol sedation (Berger et al., 2008), defects in the development of ethanol tolerance (Berger et al., 2008), and reduced voluntary consumption of ethanol (Devineni and Heberlein, 2009). Moreover, the expression of the memory gene *rut* in mushroom body (MB) neurons is necessary for robust ethanol consumption (Xu et al., 2012).

Recent studies identified new players that connect neuroplasticity and the formation of memories to the rewarding effects of ethanol intoxication. *scabrous*, which encodes a fibrinogen-related peptide that regulates Notch signaling, was shown to be necessary for the rewarding effects of ethanol intoxication (Kaun et al., 2011). Another study discovered that the sirtuin gene *Sir2 (Sirt1)*, which deacetylates histones and transcription factors, is regulated by exposure to ethanol vapor, and is required for normal ethanol sensitivity, tolerance, and for ethanol preference and reward (Engel et al., 2016).

Lastly, although this review focuses on ethanol related behaviors, it is important to mention a study that tested the role of memory mutants in nicotine-induced motor sensitivity (Hou et al., 2004). Using a startle-induced climbing assay, measuring the effect of nicotine vapor on climbing ability, Hou et al. demonstrated that *dunce* mutant flies, which harbor higher basal levels of cAMP, exhibited increased sensitivity to the depressing effects of nicotine. In contrast, DCO^H2^ (Pka-C1^H2^) and DCO^B3^ (Pka-C1^B3^) mutants that are defective in PKA showed low sensitivity to nicotine (Hou et al., 2004).

## Motivational states as an orginizing principle that shapes reward processing

Animals continuously integrate their internal physiological state with environmental signals, and subsequently choose one action over another to increase their chances of survival and reproduction. As such, the state of the organism defines which stimuli are positively reinforced, negatively reinforced or considered negligible. A classic example of this is that fruit flies have to be hungry to express appetitive memory for sugar (Krashes and Waddell, 2008; Krashes et al., 2009; Gruber et al., 2013), highlighting the ability of internal signals such as hunger to modulate learned responses of cues associated with food.

An example of the interplay between states and reward processing can also be seen in aversive conditioning in fruit flies. Pairing a neutral odor with electric shock forms an association that predicts the arrival of pain. Conversely, presenting the odor following electric shock promotes appetitive behavior, and predicts the relief of pain, implying that the end of an aversive state can also be rewarding (Tanimoto et al., 2004). This indicates that even in flies, reward is not an absolute experience, but is relative to the state in which it is perceived. Repeated stressful experiences, such as repeated exposure to heat or electric shocks, where the fly cannot evade punishment by walking away, can induce a depression-like state, leading to decline in walking activity, similar to learned helplessness paradigms in rodents (Yang et al., 2013). Uncontrollable repeated mechanical stress in flies can induce long-lasting changes in motivational states, exhibited by reduced motivation to seek rewards and reduced 5HT (serotonin) levels (Ries et al., 2017). This depression-like state can be relieved by lithium treatment or artificial activation of serotonergic neurons that project to the MB (Ries et al., 2017).

Another aspect of the interplay between motivational states and reward is the concept that different motivational states are associated with particular drives (reward seeking behavior) and specific sensory sensitivity. For instance, food deprivation and satiety affect the extent of foraging behavior and food consumption, and modulate sensory perception of food related sensory stimuli (Lee and Park, 2004; Yu et al., 2004; Wu et al., 2005; Root et al., 2011; Inagaki et al., 2012, 2014b; Marella et al., 2012; Beshel and Zhong, 2013; Wang et al., 2013; Ko et al., 2015; Jourjine et al., 2016). This is achieved by coordinated regulation of several different neuropeptide and hormonal systems that integrate nutrient signals and metabolic inputs into regulation of homeostatic drives and modulation of sensory systems (Lee and Park, 2004; Yu et al., 2004; Wu et al., 2005; Inagaki et al., 2012, 2014b; Marella et al., 2012; Gruber et al., 2013; Wang et al., 2013; Jourjine et al., 2016) (reviewed by Landayan and Wolf, 2016). This presumably occurs via the activation of specific DAN innervating the MB. For example, it was recently shown that insulin triggers the opposing functions of two neuropeptide systems: short neuropeptide F (sNPF) and tachykinin, and this in turn regulates the sensitivity toward appetitive and aversive odors (Ko et al., 2015). Serotonergic neurons were also shown to modulate motivational states that regulate feeding behavior and sugar associated reward (Burke et al., 2012; Sitaraman et al., 2012). Recently, a set of 15 serotonergic neurons was identified, that when activated, induces a fed fly to eat as if it was food deprived, and promotes the formation of appetitive memory (Albin et al., 2015). These findings imply that specific sub-populations of neurons act to shift motivational states, and thus control the way by which sensory stimuli that is associated with the experience is processed and affects behavior.

### NPF system as a molecular signature for reward states

The NPF/NPF-receptor system is emerging as a central player in modulating and encoding motivational states associated with sugar reward, sexual, and drug reward, and the homeostatic regulation of motivational responses. The activity of NPF-expressing neurons mimics a state of food deprivation, and promotes rewarding memories in satiated flies, via a subset of downstream NPF receptor expressing dopaminergic neurons that innervate the MB (Krashes et al., 2009). Additional studies revealed NPF's role in encoding other motivational aspects of feeding, such as promoting feeding (Wu et al., 2005), encoding the valence/attractiveness of food related odors (Beshel and Zhong, 2013; Beshel et al., 2017), and enhancing sugar sensitivity in sugar-sensing sensory neurons (Inagaki et al., 2014b). In addition, NPF serves as a homeostatic integration point of two interconnected systems: sleep and feeding. NPF regulates starvation, which induces sleep suppression, suggesting that the NPF system acts to encode a hunger signal that promotes an arousal state associated with high motivation to seek food (Keene et al., 2010; He et al., 2013; Chung et al., 2017).

Another example that demonstrates the interplay between motivational states and ways by which reward stimuli are perceived, is the role of NPF in integrating sexual deprivation and drug related rewards. Male flies perceive both mating interactions and ethanol intoxication as rewarding (Kaun et al., 2011; Shohat-Ophir et al., 2012). Mated male flies exhibited reduced motivation to consume ethanol containing food and have had high levels of NPF transcript, while sexually deprived male flies exhibited higher motivation to consume ethanol containing food and lower NPF transcript levels. Furthermore, activation of NPF neurons is rewarding in itself, reduces ethanol consumption, and prevents the formation of appetitive memory toward ethanol. This implies that experiences that modulate motivational states, can affect the reinforcing value of other rewarding stimuli.

The causal link between environmental stimuli, NPF levels and modulation of motivational behaviors has been documented in several studies. Reduction in NPF transcription and the activity of NPF-positive neurons was observed in response to negative environmental inputs, such as the presence of parasitic wasps and sexual deprivation, while NPF induction occurred in response to mating and ethanol intoxication (Shohat-Ophir et al., 2012; Kacsoh et al., 2013; Gao et al., 2015). Altogether, this suggests that NPF neuronal systems are central to the interplay between states and reward processing. Still, further studies are required to uncover the mechanism that connect NPF neuronal activity to activity of all specific DANs that project to the MB, and the neuronal and cellular mechanisms that allow this system to represent and affect a general reward state in the brain.

The different roles of NPF/R system in regulating motivational and homeostatic features of behavior are conserved between flies and mammals. A large number of studies demonstrate the central role of NPY (the mammalian homolog of NPF) in regulating feeding and the motivation to feed (Tatemoto et al., 1982; Clark et al., 1984; Flood and Morley, 1991; Kalra et al., 1997; Bannon et al., 2000; Day et al., 2005; Keen-Rhinehart and Bartness, 2007). A recent study uncovered a functional link between firing activities of NPY/AgRP neurons and energy homeostasis, wherein starvation induces an increase in NPY/AgRP firing rate, which in turn promotes re-feeding (He et al., 2016). The NPY system also functions in regulating sleep and wake homeostasis (Szentirmai and Krueger, 2006; Wiater et al., 2011; He et al., 2013). A study performed in zebrafish (Danio rerio) identified NPY signaling and NPY expressing neurons as regulators of zebrafish sleep, promoting sleep by inhibiting noradrenergic signaling, thus linking NPY signaling to an established arousal promoting system (Singh et al., 2017). In addition to its role in regulating natural physiological response, NPY has long been implicated in regulating drug addiction (for review on its role in ethanol addiction see (Thorsell and Mathé, 2017). NPY administration relieves the negative affective states of drug withdrawal and depression (Stogner and Holmes, 2000; Redrobe et al., 2002). Recently, a neuronal mechanism for the interplay between stress and reward systems on ethanol binge drinking was dissected in mice and monkeys, providing the first evidence for NPY and CRF functional interaction within neurons of the BNST (a limbic brain structure that is enriched with NPY and CRF neurons) (Pleil et al., 2015). Activation of the NPY Y1 receptor in the BNST led to enhanced inhibitory synaptic transmission in CRF neurons, which reduced binge alcohol drinking (Pleil et al., 2015). Their findings propose CRF neuronal function as a target for future therapies aimed to prevent and treat alcohol abuse.

## Concluding remarks

**Figure 2:**
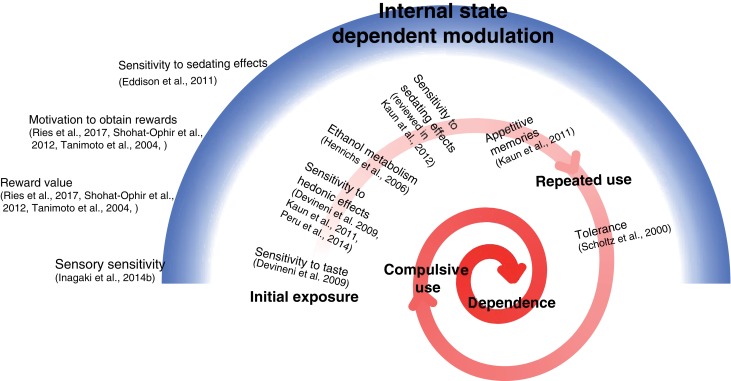
Schematic model illustrating the genetic and motivational elements that influence the likelihood of progressing from initial exposure to repeated use. Red spiral depicts the multistage progression from initial drug exposure to drug dependence and addiction and the behavioral features that are shaped by molecular and neuronal mechanisms. Blue arc depicts the way by which internal state can modulate different features in reward processing via molecular and neuronal mechanisms affecting sensory sensitivity to reward related cues, the motivation to seek and obtain rewards, and the reinforcing value of the consumed reward.

The risk of developing addiction is determined by molecular and neuronal mechanisms that influence the likelihood of progressing from initial drug exposure to repeated use. These mechanisms can shape the experience of initial consumption, the amount consumed, and the relative value of its reinforcing properties (Figure 2). For instance, genetic variations in bitter taste receptor and ethanol metabolism pathway influence the risk to develop addiction (Hinrichs et al., 2006; Yu and McClellan, 2016). Enhanced sensitivity to bitter taste is associated with reduced risk, and variations in ethanol metabolism lead to enhanced negative side effects and reduce the likelihood of repeated use, and therefore the risk to develop addiction (Figure 2). Studies in *Drosophila* demonstrated the functional link between ethanol metabolism and sensitivity to acute ethanol exposure (Ogueta et al., 2010). Other genetic components that control sensitivity to the hedonic and sedating effects of ethanol play a role in determining the extent of initial consumption and likelihood of repeated use. Upon repeated use, genetic factors that determine the extent of tolerance to ethanol-mediated responses can also shape the amount that is needed to reach the euphoric state (Figure 2).

An analogy for reward states can be proposed in which high reward state is illustrated by a full “reservoir” and low state by an empty “reservoir.” One can speculate that vulnerability to addiction is related to the size of “reservoir” to be filled (Bar, 2012). According to this model, bigger reservoir will require greater amounts of rewarding experiences in order to be filled. In addition, individuals can possess different sensitivity to fluctuations in the levels of reward within the reservoir, where sensitive individuals have increased motivation to fill up the reservoir with any type of reward, while others will be less affected by fluctuations, corresponding to reduced reward-seeking behavior.

Lastly, prior experience/motivational states can also enter into this equation, modulating different aspects of drug response. For instance, social isolation affects sensitivity to ethanol sedation (Eddison et al., 2011), pain can modulate the perception of reward-related cues (Tanimoto et al., 2004), while sexual deprivation and stress modulate the motivation to seek and obtain rewards (Shohat-Ophir et al., 2012; Ries et al., 2017; Figure 2). It is postulated that these different conditions shape the repertoire and function of proteins within neurons that mediate reward processing. As a consequence, the reward baseline is shifted, which presumably modulates the motivation to obtain rewards, the value of the consumed reward, and the likelihood to continue consuming drug rewards (Figure 2). Still, the means by which different conditions and prior experiences are encoded in the reward system and lead to changes in motivational states are largely unknown.

Recent advances in the ability to purify RNA from genetically tagged neuronal populations (Henry et al., 2012; Abruzzi et al., 2015), coupled with improvement in RNAseq technologies, make it now possible to bridge the gap between the specific transcriptomic repertoire and specific experiences/states. In this respect, it is now possible to profile the repertoire of coding mRNA, non-coding RNAs, and RNA modifications such as RNA editing, as well as the metabolome and proteome of specific neurons in every state. This can facilitate studies exploring the contributions of co-transcriptional mechanisms such as RNA editing, post-transcriptional mechanisms such as RNA methylation, and post-translational mechanisms in shaping the vulnerability to drug addiction. Further in-depth mechanistic studies will be required to connect specific regulation events to their functional relevance in shaping the transition from initial drug use to addiction.

## Author contributions

All authors listed have made a substantial, direct and intellectual contribution to the work, and approved it for publication.

### Conflict of interest statement

The authors declare that the research was conducted in the absence of any commercial or financial relationships that could be construed as a potential conflict of interest.
